# Editorial Comment: Vasectomy re-reversal: effectiveness and parameters associated with its success

**DOI:** 10.1590/S1677-5538.IBJU.2020.0310.1

**Published:** 2021-03-25

**Authors:** Rodrigo R. Vieiralves

**Affiliations:** 1 Hospital Federal da Lagoa Serviço de Urologia Rio de JaneiroRJ Brasil Serviço de Urologia, Hospital Federal da Lagoa, Rio de Janeiro, RJ, Brasil

## COMMENT

Every year 500,000 men undergo a vasectomy in the United States. Around 2% to 6% will reverse the procedure, seeking a new conception, usually as a result of a new relationship (1, 2). But, what to do when the vasectomy reversal fails? We know that the time between vasectomy and reversal surgery is crucial. The Vasovasostomy Study Group showed patency and pregnancy rates of 97% and 76%, respectively, for the 3-year obstruction interval, which dropped to 71% and 30% for the 15-year obstruction interval or more (3). Achieve a spontaneous pregnancy after a re-reversing the vasectomy is something challenging and that’s exactly what the article in question is about. In these cases, in which the reversal fails and the patient seeks spontaneous pregnancy, the only way is a vasectomy re-reversion, a new microsurgical procedure performed after the first reversal. In this topic, aiming to offer quality information leading to the best clinical decision, what are the parameters capable of influencing the success rate of a vasectomy re-reversal (4)? We will see below.

There is time that the scientific community has concerned if microsurgical vasovasostomy and vasoepididymostomy provided satisfactory patency rate and natural pregnancy rates for patients with a previous failed vasectomy reversal (5). In this interesting pleasant reading article, the authors raise a topic that still lacks robust data: Vasectomy re-reversion (6). A first interesting aspect that worth mentioning is the parameters used to define the reversal failure. The authors were careful to highlight that the failure is defined as a persistent postoperative azoospermia, or a good sperm concentration with very little or even no motility and / or associated with necrozoospermia. This is important because the final and true desire is conception and, therefore, these criteria are of great importance when we consider the functionality of the sperm resulting from re-reversion. Using a simple and clear methodology, the authors present the study with a total of 18 patients, with the objective of determining the factors associated with the success of the vasectomy re-reversion. For this, the patient´s age at the time of the reversion, time between the vasectomy and the vasectomy reversal, time between the vasectomy reversal and the vasectomy re-reversal, the presence of sperm in the proximal deferens fluid and results of seminal analysis after vasectomy re-reversion were analyzed.

Before we reach the important conclusions, some aspects need to be highlighted, seeking to clarify other aspects involved in the reversal or re-reversion of the vasectomy. The first would be the surgeon’s experience. The greater the number of procedures, the greater the success rate. We felt the absence of comments in this regard. Another important aspect is about the chosen technique: the authors made it clear in the methodology that the suture was performed in a single plane with 9-0 nylon but nothing was treated about the number of “stitches”. Most surgeons today choose 4 single layer sutures and as we know this aspect can impact local healing and post-operative patency rates. Still, as limiting factors, we highlight that low number of patients, a limiter to more robust conclusions. In addition, despite the author´s elegant justification during the discussion, the pregnancy rate is not mentioned, which is the main expected result and major outcome after vasectomy reversal. Several other factors, such as clinical conditions associated with infertility were also not mentioned and may be a hidden confounding factor, nothing that takes away the relevance of the present work.

Focusing into the target of the study, we observe in this excellent paper a clarifying study format about the factors associated with the presence of seminal quality parameters in the ejaculate after the vasectomy re-reversion. The authors confirm, corroborating with the literature, that among the factors studied, the intraoperative identification of the presence of sperm in the proximal vas deferens is the factor that most correlates with a favorable prognosis demonstrating the importance of collecting this material during the surgery. In the future, more studies like this may gradually guide us about the best conduct, allowing us to offer our patients quality information about the most appropriate way to achieve pregnancy, respecting the couple’s desire.

## References

[1] 1. Goldstein M. Vasectomy reversal. Compr Ther. 1993;19:37-41. 8334860

[2] 2. Pinto LOAD, de Barros CAV, de Lima AB, Dos Santos DR, de Bacelar HPH. Portable model for vasectomy reversal training. Int Braz J Urol. 2019;45:1013-9. 10.1590/S1677-5538.IBJU.2019.0092 PMC6844345 31268638

[3] 3. Belker AM, Thomas AJ Jr, Fuchs EF, Konnak JW, Sharlip ID. Results of 1,469 microsurgical vasectomy reversals by the Vasovasostomy Study Group. J Urol. 1991;145:505-11. 10.1016/s0022-5347(17)38381-7 1997700

[4] 4. Fox M. Failed vasectomy reversal: is a further attempt worthwhile using microsurgery? Eur Urol. 1997;31:436-40. 10.1159/000474503 9187904

[5] 5. Hollingsworth MR, Sandlow JI, Schrepferman CG, Brannigan RE, Kolettis PN. Repeat vasectomy reversal yields high success rates. Fertil Steril. 2007;88:217-9. 10.1016/j.fertnstert.2006.11.077 17336963

[6] 6. Lorenzini MS, Lorenzini F, Bezerra CA. Vasectomy re-reversal: effectiveness and parameters associated with its success. Int Braz J Urol. 2021;47:544-48. 10.1590/S1677-5538.IBJU.2020.0310 PMC7993950 33621001

